# The Hydration Structure at Yttria-Stabilized Cubic Zirconia (110)-Water Interface with Sub-Ångström Resolution

**DOI:** 10.1038/srep27916

**Published:** 2016-06-15

**Authors:** Binyang Hou, Seunghyun Kim, Taeho Kim, Jongjin Kim, Seungbum Hong, Chi Bum Bahn, Changyong Park, Ji Hyun Kim

**Affiliations:** 1 High Pressure Collaborative Access Team, Geophysical Laboratory, Carnegie Institution of Washington, Argonne, IL 60439, USA; 2 Department of Nuclear Science and Engineering, School of Mechanical and Nuclear Engineering, Ulsan National Institute of Science and Technology, Ulsan 44919, South Korea; 3 Materials Science Division, Argonne National Laboratory, Argonne, IL 60439, USA; 4 Department of Materials Science and Engineering, KAIST, Daejeon 34141, South Korea; 5 School of Mechanical Engineering, Pusan National University, Pusan 46241, South Korea

## Abstract

The interfacial hydration structure of yttria-stabilized cubic zirconia (110) surface in contact with water was determined with ~0.5 Å resolution by high-resolution X-ray reflectivity measurement. The terminal layer shows a reduced electron density compared to the following substrate lattice layers, which indicates there are additional defects generated by metal depletion as well as intrinsic oxygen vacancies, both of which are apparently filled by water species. Above this top surface layer, two additional adsorbed layers are observed forming a characteristic interfacial hydration structure. The first adsorbed layer shows abnormally high density as pure water and likely includes metal species, whereas the second layer consists of pure water. The observed interfacial hydration structure seems responsible for local equilibration of the defective surface in water and eventually regulating the long-term degradation processes. The multitude of water interactions with the zirconia surface results in the complex but highly ordered interfacial structure constituting the reaction front.

Zirconia, ZrO_2_, has numerous applications, e.g., for gas sensing^1^, solid oxide fuel cell electrode^2^,^3^, bio-medical materials^4^, to name a few, and also plays a critical role to protect zirconium alloy surface in highly corrosive environment, e.g., zirconium alloy cladding in pressurized water reactor^5^. The oxide degradation and the continuous growth of passive layer are detrimental to the materials integrity, which are directly attributed to the interaction of the surface with water. Thus, knowledge of the detailed interfacial structure such as interfacial hydration, surface relaxation and adsorption of ions is of fundamental importance for understanding the degradation mechanism.

For many decades, researchers have studied the degradation of zirconia in relation to the phase transformation. Kobayashi *et al*.^6^ first reported the transformation of tetragonal to monoclinic structure after heating above 1,400 °C for several hours. It was also shown that chemically stabilized zirconia (3% mole Y_2_O_3_ in ZrO_2_) undergo hydrothermal degradation from tetragonal to monoclinic in humid condition even at temperatures as low as 100 °C^7^. A higher dopant rate of cubic phase yttria-stabilized zirconia (YSZ) also showed phase transformation at 250 °C under humid conditions for extended period of time (i.e., over 2 years)^8^. Recently, Kim *et al*. used *in-situ* Raman spectroscopy to confirm the phase transformation of oxide film on zirconium alloy surface under hydrothermal conditions^9^.

In addition, there has been a great deal of effort to the study of interfacial structures of zirconia in contact with water, attempting to reveal the oxide degradation mechanism from the molecular insight. It is known that water molecules are adsorbed on zirconia surface inducing new OH groups, which remains on the surface as point defects but eventually migrates into the substrate lattice to initiate the degradation process^10^,^11^,^12^. Water is chemisorbed dissociatively on surfaces of zirconia, revealed by gas chromatography/mass spectrometry^13^. Dissolution of the oxide layer on zirconium surface is also suspected as a possible breakdown mechanism under high temperature condition^14^. A first-principle study of the hydroxylation of zirconia surface suggests generally more favorable adsorption of dissociative water than molecular water on zirconia surface, especially, at low coverage^15^. Dynamics of the surface water molecules studied by a quasi-elastic neutron-scattering technique, however, suggests co-existing water modes on the ZrO_2_ surface in addition to the surface hydroxyl OH groups^16^,^17^. Inelastic neutron and Raman scattering studies also show the existence of chemisorbed hydroxyl groups and physisorbed water simultaneously on surface of ZrO_2_ nanoparticles^18^,^19^.

Similar mechanisms on YSZ in contact with water were also studied. Yoshimura *et al*. proposed a degradation process of YSZ by H_2_O to form OH^−^ on the surface which initiates the stressed sites^20^. Raz *et al*. also studied the adsorption of water layers on YSZ and suggested the first water layer to be chemisorbed on the Zr sites and the second layer to be physisorbed on top of the chemisorbed water layer^21^. The cubic form of YSZ were also found to form hydroxyl groups on the surface^22^. Degradation during aging of transformation-toughened YSZ at 250 °C was reported, caused by depletion of dopant metal when exposed to water vapor^23^. Costa *et al*. studied the energetics of water adsorption on YSZ surfaces to reveal the reduction of anhydrous surface energy by adsorbing water on the surface^24^.

These studies of zirconia-water interfaces have brought into great knowledge about the initiation mechanism of the degradation and corrosion processes. The results can be summarized into two main opinions: firstly, the degradation process under humid conditions starts from the water chemisorption on the surface Zr sites, which creates point defects; secondly, the degradation process involves the leach of the dopant metal ions, which makes the crystal structure unstable and therefore undergoes phase transformation. Despite the extensive studies, these two opinions hardly found their consensus^25^, suggesting the lack of knowledge on the experimentally determined structure of the zirconia-water interface at the molecular scale.

Here we use the high-resolution X-ray reflectivity (HRXR) to determine the interfacial hydration structure at YSZ (110)-water interface with atomic scale resolution. Over the past three decades, this synchrotron-based X-ray reflectivity technique has emerged and been developed into a very sophisticated and cutting-edge method for probing the interfacial structures with sub-Ångström resolution^26^,^27^,^28^,^29^,^30^,^31^,^32^. Detailed hydration structures and molecular level processes at various mineral-water interfaces have been studied with this technique. We utilize a single crystal YSZ substrate because the HRXR technique requires an atomically flat and homogenous solid surface for allowance of measuring the intensities consistently over a wide range of reflection angles. The nearly perfect single crystal YSZ substrates are commercially available, while zirconia crystal is only stable in monoclinic form at T < 1,170 °C^33^ and the high purity single crystal zirconia is difficult to obtain from melt due to its large volume expansion from tetragonal to monoclinic transformation^34^. Therefore, we take an advantage of YSZ’s crystallinity to bypass those difficulties and at the same time exploit its own merit as a model system representing defective zirconia surface.

The unit cell of cubic YSZ along (110) direction exhibits tetragonal structure (*a* = *c* = 3.642 Å and *b* = 5.152 Å). Figure 1 shows the crystal structure of the cubic ZrO_2_ in (110) direction from both top and side views. There is only one physically possible termination of the surface in this particular orientation, where both metal and oxygen species are exposed. In this study, we measure the HRXR from the YSZ (110)-water interface at ambient condition with a specular *θ-*2*θ* scan geometry. The schematic illustration of the thin film sample cell in use and the experimental geometry for the X-ray reflectivity measurement are depicted in Fig. 2. We performed a non-linear least squares fitting to the experimental data to derive the electron density profile along the surface normal direction. The detailed structure including the termination of the YSZ (110) surface and the characteristic interfacial hydration was obtained. Our result reveals multitude of water interaction with the surface: filling the vacancies from intrinsic oxygen deficiency and surface metal depletion, locally hydrating and stabilizing surface metal species, and spatially confining the interfacial hydration process within a few molecular layers of water resulting in a highly ordered interfacial structure.

## Results

### High-resolution X-ray reflectivity

Figure 3 shows the measured specular HRXRs and their best-fit results for two different YSZ (110) substrates (Sample #1 and Sample #2) in contact with water as a function of momentum transfer *Q*_z_ = 4*π*sin*θ*/*λ*, where *θ* is the X-ray incident angle, and *λ* is the wavelength. The measured data spans from *Q*_z_ = 0.70 through 5.95 Å^−1^. Since the Bragg reflection of face-centered cubic (110) is forbidden^35^, the first Bragg peak corresponds to the (220) refection at *Q*_z_ = 2*π/d*_220_ = 3.45 Å^−1^. The spatial resolution in the corresponding real-space structure is determined by the maximum *Q*_*z*_, which gives an FWHM resolution of *π/Q*_z, max_ = 0.53 Å assuming a Gaussian distribution. Compared to Sample #1, the reflectivity data from Sample #2 appears to have lower mid-zone intensities. This behavior is observed when the fractional occupancy of adsorbed layer at the interface is different^31^. The detailed quantitative analysis based on the interfacial structure factor model is given in the following sections.

We performed Atomic Force Microscopy (AFM) measurement to test the surface morphologies and roughness (Fig. S1). The AFM measurement confirms that the two samples have certainly different morphologies but commonly lack the distinct steps, kinks, and ledges on the surface. The root-mean square (r.m.s.) roughness is 2.4 Å for Sample #1 and 1.0 Å for Sample #2, respectively. Considering the physical unit cell height (*d*_220_ = 1.82 Å), the both samples show quite smooth surface but with extended non-uniformity without distinct lateral boundaries (see Supplementary Information).

### Structure factor analysis and the best-fit result

The detailed description of the HRXR analysis for solid-water interface can be found in the review articles^30^,^31^. The measured reflectivity can be reproduced by a structure factor model based on the known crystal structure of solid substrate and a few modeling strategies for the interface:


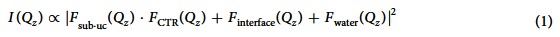


Although the structure factor model (Eq. (1)) consists of three terms, it represents four major physical components from the solid-water interface: (1) the semi-infinite single crystal, (2) the near surface unit cells which deviate to any extent from the bulk, e.g., relaxation in the *d*-spacing, depletion, etc., (3) the adsorbed layers containing water and other species such as ions, and (4) the semi-infinite bulk water. In Equation (1), *F*_sub·uc_(*Q*_z_)·*F*_CTR_(*Q*_z_) corresponds to component (1), *F*_interface_(*Q*_z_) includes both (2) and (3), and *F*_water_(*Q*_z_) represents component (4). The intensities measured at the mid-zone regions between Bragg peaks are more sensitive to the interfacial structure, while the near-Bragg regions are more influenced by the single crystal substrate. The interfacial structure can be determined through non-linear least squares fitting to the measured X-ray reflectivity data using a set of model structure factor parameters. The electron density profile for each scattering atom is modeled by a Gaussian distribution, whose FWHM defines the spatial resolution. The experimental resolution, determined by the maximum measured *Q*_z,max_, is convoluted to the total electron density profile when it is reconstructed based on the best-fit model. The exact expressions and the detailed modeling strategies can be found in SI.

In Fig. 3, the best-fit results reproduce both measured X-ray reflectivity data very well. The chi-square, defined as *χ*^*2*^ = *∑*_*i*_(*I*_*calc.,i*_ − *I*_*meas.,i*_)^2^/[*σ*_*i*_^*2*^(*N−P*)] where *I*_*calc.,i*_ is the calculated intensity, *I*_*meas.,i*_ is the measured intensity, *σ*_*i*_ is the standard error of the measured data, *N* is the number of data points, and *P* is the number of parameters, respectively^36^, from the least-squares fitting is 1.159 and 1.398 for Sample #1 and Sample #2, respectively. The electron density profiles along the surface normal direction are then derived based on the best-fit parameters (listed in Table S1 in SI). Figure 4a shows the ball and stick model of the atomic structures depicting the most-likely scenario for the interfacial hydration processes and Fig. 4b is the corresponding electron density profiles (black line for Sample #1 and red dashed line for Sample #2). The profiles represent the laterally averaged electron densities.

Despite the distinct difference observed in the measured HRXR spectra, the resultant interfacial structures as shown by electron density profiles (Fig. 4b) are surprisingly consistent with each other except for relatively higher total density at the position of second adsorbed layer for Sample #1, which should be correspondingly responsible for the promoted intensities at the mid-zone. However, according to the estimated values and errors listed in Table S1, the difference is indistinguishable within the errors. It is true not only for the particular layer parameters but also for all other parameters. The model parameters were chosen to reproduce both of the measured data with an identical definition and the minimum required number of parameters to avoid large covariance between the parameters. Therefore, the qualitatively consistent features (Fig. 4b) observed from two independent measurements, which show statistically significant differences (Fig. 3), are meaningful to confirm the general characteristics of the YSZ (110)-water interface structure. Other sets of parameters specific to each sample were also tested, which reproduced similar total electron density profiles.

The two samples show similar hydration structures with an adsorbed water layer overlapping the terminal layer (at z = 0 in Fig. 4) followed by the two additional layers before connected to the bulk water. The surface roughness of these two samples, as calculated from the estimated Robinson factors^26^, turned out to be very close to each other, 1.34 Å and 1.38 Å for Sample #1 and Sample #2, respectively. These values are different from the r.m.s ones obtained by the AFM measurements, which suggests that the general characteristics for YSZ (110)-water interface structure observed in this study are independent of the surface morphology.

### Interfacial structure

For the surface relaxation, the best-fit results show alternating positive and negative relaxations in the lattice parameters of the top five surface unit cells (*a*_1_ through *a*_5_, Table S1). However, the variations are negligibly small, less than 0.01 Å displacement from the bulk unit cell lattice constant (<0.3% uniaxial strain per each unit cell). Compared to an *ab-initio* calculation result which reports ~0.2 Å top surface relaxation followed by 0.09 Å next layer for anhydrous YSZ (110) surface^37^, the observed relaxations are remarkably small.

At the terminal layer of substrate (*z* = 0), the metal occupancy is found to be 0.728 ± 0.063 for Sample #1 (~27% depletion from the ideal occupancy 1) (Table S1 in SI). The position of O at the top surface is found to be slightly displaced from that of the metal atoms at *Δy *= −0.007 ± 0.052 Å. The best-fit results show also a layer of O equivalent (i.e., estimated by an oxygen occupancy), vacancy-filling water overlapping the terminal layer (dashed blue line in Fig. 4b) with distribution width of 0.132 ± 0.047 Å. The detailed explanation for the fitting parameters to represent these highly overlapping features at the same position is given in SI.

Above the terminal layer, there are two additionally adsorbed layers found at *z*_1_ = 1.687 ± 0.048 Å (dashed gold line) and *z*_2 _= 3.439 ± 0.118 Å (dashed purple line), respectively. The first adsorbed layer has abnormally high density to be a pure water layer; therefore, it probably includes higher density species such as metal atoms. The second adsorbed layer is presumably pure water layer as its density is comparable to that of pure water. The interfacial hydration structure is then followed by the sharp first layer of bulk water at 4.918 ± 1.019 Å above the substrate surface (or 1.479 Å above the 2^nd^ adsorbed layer) and quickly damps into the featureless bulk structure^31^,^38^ (dashed green line in Fig. 4b).

When characterizing the adsorbed hydration structures next to the terminal layer, we initially assumed pure water for these layers. The best-fit results, however, yielded unphysically dense first adsorbed layer as a pure water layer, 2.65 times of the bulk water density (at *z*_1_ = ~1.7 Å in Fig. 4b). This result suggests that this hydration layer include additionally heavier species than water such as Zr or Y atoms, or both. Each metal atom has about 4 times electrons (40 or 39) of water (10), therefore, 2.65 times of bulk water density corresponds to only a fraction (~0.66) of metal occupancy in this layer. The unit cell has an occupancy of 1 metal and 2 oxygen atoms (total 3 atoms) on each plane; therefore, there is still enough space for water molecules to fill in in this layer. Consequently, the metal atoms in this layer are likely surrounded by water molecules, with the possibility of forming extra M-O bonding with the substrate surface O atom and water in the 2^nd^ adsorbed layer in its three dimensional configuration. We modeled them as a hydrated metal species, [M(H_2_O)_n_]^*x*+^, where n represents the hydration shell of the metal species, and *x* represents the possible valence of the hydrated complex. The inclusion of the metal ions in the first adsorbed layer turned out to reproduce the measured data very well. However, due the similar electron numbers of Y and Zr, modeling based on either complex turned identical. Table S1 reports one of the tested models as a representative for simplicity.

Sample #2 results reproduce the qualitatively same features as described in the previous section. The following discussions are based on Sample #1 results.

## Discussion

At the terminal layer, the metal depletion, as observed (~27%), should result in a substantial increase in the number of vacancies in addition to the inherent vacancies^39^. These vacancy sites, regardless of their origins, are expected to be occupied by “vacancy filling” water^40^ and they are likely to be dissociated based on numerous pre-existing contexts^10^,^11^,^12^,^13^,^15^,^16^,^17^,^18^,^19^,^21^. A recent density functional study supports that, although along the different orientation of (111), water molecules are expected to be strongly chemisorbed on the YSZ surface, too^41^. Therefore, it is reasonable to postulate that at least a portion of this vacancy filling water (blue dashed line in Fig. 4b) should be dissociative and chemisorbed on the surface to form hydroxide. The best-fit value for the distribution width of this layer is 0.132 ± 0.047 Å (Table S1), which indicates quite narrowly confined geometry for the adsorption. It is also worth pointing out that the metal depletion is limited on the top surface layer in our model. The water species confined in this layer may be related to the process relevant to reducing the interfacial energy relative to the anhydrous surface^24^, although the role of other two adsorbed layers should be considered together.

**Figure 1:**
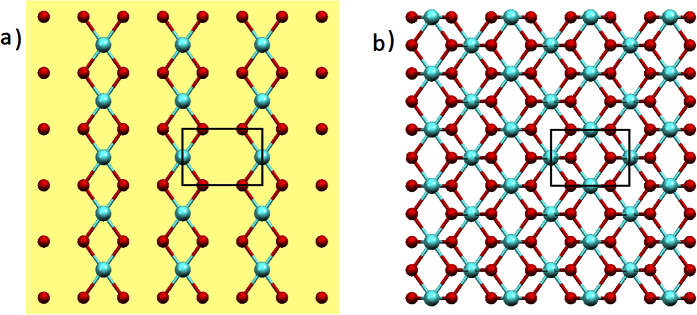
Crystal structure of cubic ZrO_2_. (**a**) Top view of (110) surface and (**b**) side view with indication of the unit cell redefined for this particular orientation (solid rectangle). Teal spheres represent metal atoms, and red spheres oxygen atoms. With 8% mole Y_2_O_3_ doping, the metal atom sites consist of 14.8% Y and 85.2% Zr atoms, and the oxygen sites have occupancy of 96.3%.

The origin of the suspected metal species in the first adsorbed hydration layer lies in two possibilities. Firstly, it could come from re-adsorption of dissolved Y from the substrate. The leaching of Y from the substrate^42^ would generate a pair of freed Y^3+^ and a negatively charged vacancy left behind. Although the vacancy could be filled by water molecule, its valence could be only reduced to −2 at most as the water could only be positively monovalent (in the form of hydronium H_3_O^+^). Therefore, there will be still a relatively strong electrostatic interaction between the Y^3+^ and the negative vacancy site, which will greatly restrain the Y^3+^ from leaving the substrate surface. In addition, the formation of hydration structure and the possible Y-O bonding with surface O atoms would help greatly in reducing the surface energy to keep this configuration stable. The green arrow and orange line in Fig. 4a indicate these two processes. Secondly, the observed layer could be simply an “adlayer” or partial layer as another form of defect, which, different from what its name implies, might be produced by an extended depletion in lateral direction. As the crystalline layer for this particular orientation is a composite metal-oxygen layer allowing only one possible termination (Figs 1 and 4b), these two possibilities are undistinguishable. So the identity of metal species in this layer still remains unclear. The position of this layer (1.687 Å, Table S1) is, however, significantly lower compared to the crystalline layer spacing (1.821 Å). It is rather closer to the layer spacing of tetragonal ZrO_2_ in (100) orientation (1.793 Å)^43^, which has the same coordination characteristics with that of YSZ (110). Because of this large discrepancy from both crystalline layer spacing, it is not quite strongly supported that this layer is from crystalline adlayer. Nevertheless, the observed interfacial structure is stable at least for the short experimental time period of ~8 hrs (the experimental duration).

Immediately above the dense 1^st^ adsorbed layer, the best-fit results show an additionally adsorbed water layer with occupancy of 1.441 ± 0.347. The existence of the ordered second hydration layer is qualitatively consistent with the basic assumption that the vacancy filling by water and the metal-hydration interactions at the interface should strongly constrain the next layer hydration (see Interfacial Structure Factor Modeling Strategies in SI). Compared to the natural olivine (010)-water interface^40^, the first adsorbed layer at the YSZ (110)-water interface even includes heavier metal species besides water so that the different chemistry effect must be considered. Nevertheless, the existence of the sharp second hydration layer is characteristic to the interfacial hydration structure of our system. In Fig. 4b, the total density of this layer looks slightly higher than that expected for the normal water, although the estimated water occupancy is slightly less than expected (~1.876 per 18.76 Å^2^ for bulk water). The apparently promoted electron density seems due to its highly ordered but overlapping neighboring layers which have wide distribution widths (purple dashed line in Fig. 4b).

The first layer of bulk water (green dashed line in Fig. 4b) is followed by nearly featureless bulk water next to it, restoring the bulk water structure immediately. It is worth remarking that the transition from highly ordered interfacial water to disordered bulk water takes only one transitional layer. And also, the large overlapping between the ordered adsorbed layer and the transitional bulk water layer (i.e., the first bulk water layer) indicates a degenerate portion of the layered adsorbed water (the 2^nd^ adsorbed layer) and this transitional bulk water.

In conclusion, the structure of YSZ (110)-water interface was determined with high-resolution X-ray reflectivity. The best-fit structure suggests that the surface relaxation is negligible while the terminal layer, metal and oxygen terminated, includes a significant number of point defects indicated by the reduction of total electron density. The intrinsic oxygen vacancy sites and those generated by metal depletion are expected to be filled by water with significant amount of water being dissociative. Two distinct adsorbed layers on top of the terminal layer further characterize the interfacial hydration structure. The first adsorbed layer likely includes metal species, while the second adsorbed layer consists of pure water. There have been numerous studies showing the water chemisorption on zirconia surfaces as an important primary process for the degradation. Our study provides the detailed geometric confinement and how they are correlated with the molecularly adsorbed water. The entire interfacial hydration process is spatially confined within three molecular layers of water resulting in highly ordered layer structure. Therefore, our results suggest a possibility that a stabilization of this characteristic interfacial hydration structure, e.g., by tweaking water chemistry, would mitigate further degradation. This possibility gives us a unique opportunity to reconsider the passivation mechanism and a feasible route to control the process ultimately. The detailed elemental profile with quantitative coverage information is desired to investigate this possibility further.

## Methods

### Materials

Single crystal yttria-stabilized cubic zirconia (8% mole Y_2_O_3_), or YSZ, substrates were purchased from MTI Corporation in the dimension of 10 × 10 × 0.5 mm^3^. The orientation accuracy as estimated during the HRXR measurement is ~0.9° and the r.m.s. surface roughness is 2.4 Å for Sample #1 and 1.0 Å for Sample #2, respectively, as characterized by an atomic force microscopy (see SI). The crystal system is CaF_2_ type face centered cubic. We determined the lattice constant, 5.152 ± 0.010 Å, during our HRXR measurement. Along the surface normal direction, [110], the surface unit cell exhibits a tetragonal structure (Fig. 1). The *d*-spacing of surface unit cell reduces to 1/√2 of the original unit cell, i.e., *d*_110_ = 3.642 Å. In this paper we refer the “unit cell” to this surface unit cell and “occupancy” refers to per unit cell area of 18.76 Å^2^ (*a* = 5.152 Å × b = 3.642 Å). The unit cell consists of a fraction of ½ Zr and 1 O atoms at *z* = 0 plane, 1 Zr and 2 O atoms at *z *= 1/2*d*_110_ plane, and ½ Zr and 1 O atoms at *z* = *d*_110_ plane. Along z direction, the stacking of unit cell results in periodic arrangement of 1 Zr and 2 O atoms on each plane. Therefore, there exists only one possible termination surface (Fig. 1).

### Sample preparation

**Figure 2:**
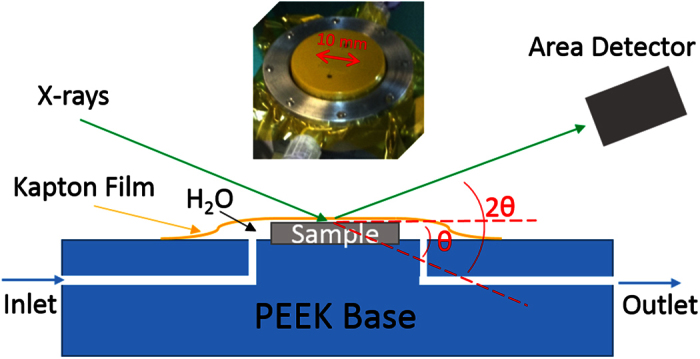
Schematic illustration of the experiment. The thin film cell for *in-situ* measurement of X-ray reflectivity from solid-water interface and the experimental geometry. The inset is a picture of an actual sample cell with sample length indicated.

**Figure 3:**
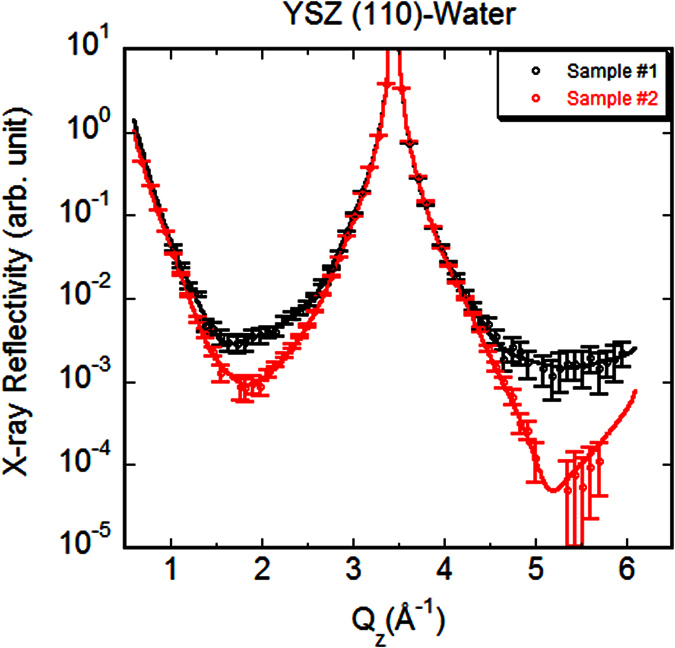
High-resolution X-ray reflectivity. Measured data (circles with error bars) from two different samples and their best-fit results (solid lines) for YSZ (110)-water interface. The intensities are scaled to match at the near Bragg peak intensities.

**Figure 4:**
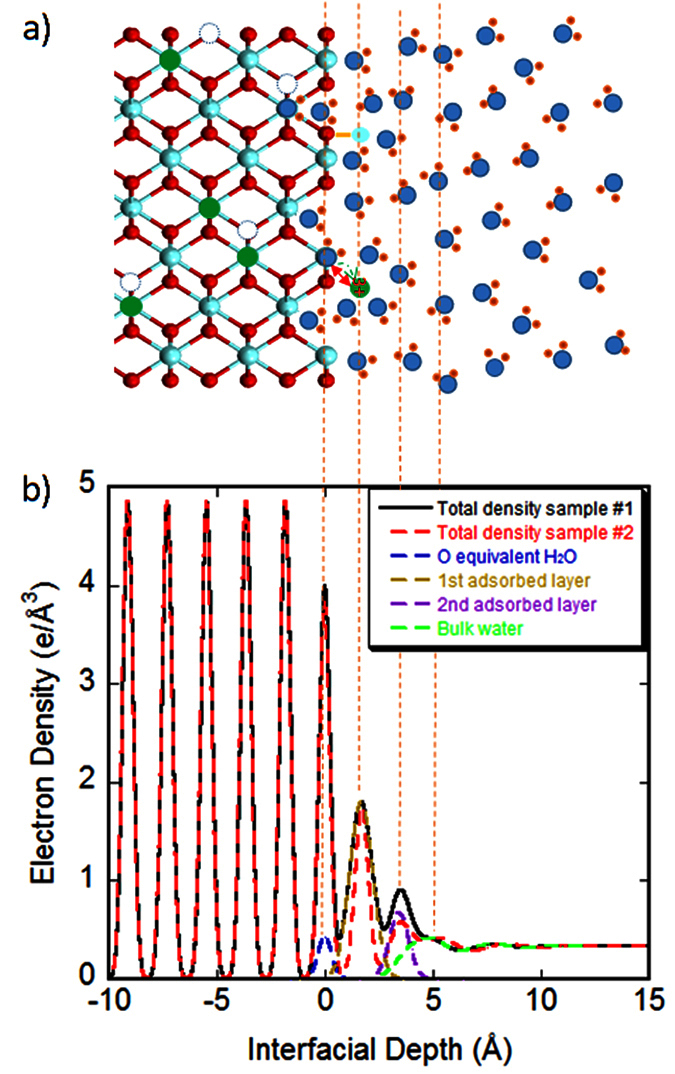
Interfacial structure and most-likely scenario of interfacial processes. (**a**) Ball and stick model of the atomic structures depicting the most-likely scenario of the interfacial hydration processes: blue filled circles with two red ear small circles represent water molecules, green filled circles represent yttrium, teal filled circles represent zirconium, red filled circles represent oxygen, and unfilled circles represent oxygen vacancies in the bulk. The green arrow shows the possible Y dissolution and re-adsorption process, and the orange line indicates M-O bonding; and (**b**) the electron density profile derived based on the best-fit parameters estimated to reproduce the measured HRXR data. The sub-profiles are expressed only for Sample #1 for clarity.

The substrate was cleaned ultrasonically in acetone, methanol and deionized water (DIW) for >15 min each successively to remove any possible organic contaminations before use. At the final stage of cleaning, the sample was extensively cleaned in more than 300 mL DIW with sonicating to remove remnant methanol on surface, which is known to be strongly adsorbed on zirconia surface. The cleaned substrate was then mounted in a thin-film cell designed for *in-situ* HRXR measurement from a solid-liquid interface (Fig. 2). The sample was covered and held in place by an 8-μm thick Kapton film on a plastic (PEEK) cell base. The Kapton cover was inflated by purging DIW to fully wet the substrate surface and the cell was repeatedly flushed to completely remove air bubbles and other remnant, if any. The cell was then naturally drained by the gravitational force to form a thin water film (less than a few tens of microns) between the YSZ (110) surface and the Kapton film for the HRXR measurement. The cell was then sealed by close valves during the measurement without water flowing through it hence remained as an isolated state at room temperature.

### High resolution X-ray reflectivity

The HRXR measurement for Sample #1 was carried out at the 33-ID-D beamline of Advanced Photon Source (APS) at Argonne National Laboratory (ANL). The incident X-ray energy was 21.000 keV, the beam was focused with Kirkpatrick-Baez mirrors to 0.05 mm (v)  × 0.2 mm (h), and the incident flux was ~10^11^ photons/sec. The HRXR for Sample #2 from a different batch was carried out at beamline 9C of Pohang Light Source (PLS) at Pohang Accelerator Laboratory (PAL) in South Korea. The incident X-ray energy was 15.000 keV. The beam was collimated to 0.1 mm (v)  × 0.7 mm (h), and the incident flux was ~1.1 × 10^11^ photons/sec. A Pilatus 100K detector, mounted on a 4-circle diffractometer arm, was used to collect the data. The scattering area on the detector was defined by a set of detector slits. The HRXR from YSZ (110)-water interface was measured in a specular *θ-*2*θ* scan geometry. The data reduction from the area detector followed the description by Fenter *et al*.^44^. A set of guard slits were used for the reduction of background scattering. The measured intensities were normalized by an incident beam monitor. The errors are propagated from the counting statistics.

### Atomic force microscopy

The Atomic Force Microscopy (Asylum Research MFP3D, Santa Barbara, CA) was used to examine the surface morphologies of the samples. The AFM was operated in contact mode to ensure atomic level resolution for probing the height distribution. The loading force was 81.4 nN and the scan rate was 0.8 Hz.

### X-ray photoelectron spectroscopy (XPS)

The XPS measurements were carried out with a Thermo Scientific K-Alpha X-ray Photoelectron Spectrometer. The first XPS measurement was done on the as-cleaned single crystal substrate and then followed by several measurements after sputtering time of 2, 4, 6 and 20 seconds, respectively. The binding energies were calibrated with the adventitious C 1*s* peak at 284.6 eV.

## Additional Information

**How to cite this article**: Hou, B. *et al*. The Hydration Structure at Yttria-Stabilized Cubic Zirconia (110)-Water Interface with Sub-Ångström Resolution. *Sci. Rep.* **6**, 27916; doi: 10.1038/srep27916 (2016).

## Supplementary Material

Supplementary Information
