# Supplementary material for: The Hydration Structure at Yttria-Stabilized Cubic Zirconia (110)-Water Interface with Sub-Ångström Resolution
Source: Sci Rep. 2016 Jun 15;6:27916. doi: 10.1038/srep27916 (PMC4908582; doi:10.1038/srep27916)
Supplement: Supplementary Information [file srep27916-s1.pdf]

Supplementary Information:

The Hydration Structure at Yttria-Stabilized Cubic Zirconia (110)-Water Interface with Sub-Ångström Resolution

Binyang Hou, Seunghyun Kim, Taeho Kim, Jongjin Kim, Seungbum Hong, Chi Bum Bahn, Changyong Park & Ji Hyun Kim

Structure Factor Model

The reflectivity measured in a specular geometry can be reproduced from a structure factor model based on the known crystal structure of solid substrate and a few modeling strategies for the interfacial structures:

$$I(Q_z) = S\cdot\left(\frac{4\pi r_e}{Q_z A_{\mathrm{uc}}}\right)^2 \cdot T_{\mathrm{cell}}(Q_z)\cdot|B(Q_z)|^2\cdot|F_{\mathrm{sub\cdot uc}}(Q_z)\cdot F_{\mathrm{CTR}}(Q_z)+F_{\mathrm{interface}}(Q_z)+F_{\mathrm{water}}(Q_z)|^2 . \quad \text{(S1)}$$

Q_z is the momentum transfer, $4\pi\sin\theta/\lambda$, where θ is the X-ray incident angle, and λ is the wavelength. S is a scale factor. r_e=2.8179×e^{-15} m is the classical electron radius. A_{uc} is the surface area of unit cell. $T_{\mathrm{cell}}(Q_z)$ is the thin-film cell geometry correction factor for X-ray attenuation as a function of incident angle with given thickness of water D_w and X-ray attenuation length of water Λ:

$$T_{\mathrm{cell}}(Q_z)=\exp(-8\pi D_w/Q_z\lambda\Lambda). \quad \text{(S2)}$$

$|B(Q_z)|^2$ is the effect of surface roughness on the reflectivity as expressed with Robinson β factor in terms of Q_z and d-spacing of the unit cell d_{spacing}[1]:

$$|B(Q_z)|^2 = \frac{(1-\beta)^2}{1+\beta^2-2\beta\cos(Q_z d_{\mathrm{spacing}})}. \quad \text{(S3)}$$

$F_{\mathrm{sub\cdot uc}}(Q_z)\cdot F_{\mathrm{CTR}}(Q_z)$ corresponds to the semi-infinite single crystal substrate structure factor:

$$F_{\mathrm{sub\cdot uc}}(Q_z) = \sum_j^{\text{unit cell}} Occ_j f_j \exp(iQ_z z_j)\exp\left(-\frac{1}{2}Q_z^2 u_j^2\right), \quad \text{(S4)}$$

$$F_{\mathrm{CTR}}(Q_z) = \frac{1}{1-\exp(-iQ_z d_{\mathrm{spacing}})}, \quad \text{(S5)}$$

where Occ_j, f_j, z_j, and u_j are the occupancy, atomic scattering factor, position, and thermal vibrational amplitude of j-th atom in the unit cell, respectively. $F_{\mathrm{interface}}(Q_z)$ includes the near

surface unit cells which deviate to any extent from the bulk, e.g., relaxation in the d-spacing, depletion, etc., and the adsorbed layers containing water and other species such as ions, which can be expressed by the same form used in the equation (S4). The interfacial structure factor sums over all species including atoms in the relaxed surface unit cells, adsorbed water, ions and so on:

$$F_{\text{interface}}(Q_z) = \sum_{j}^{\text{interfacial species}} Occ_j f_j \exp(iQ_z z_j)\exp(-\frac{1}{2}Q_z^2 u_j^2) \ . \quad \text{(S6)}$$

$F_{\text{water}}(Q_z)$ corresponds to the semi-infinite bulk water:

$$F_{\text{water}}(Q_z) = \frac{f_{H_2O}\exp(-\frac{1}{2}Q_z^2\sigma_{0,\,B}^2)\exp(iQ_z z_{0,\,B})}{[1-\exp(-\frac{1}{2}Q_z^2\sigma_{\text{avg.,B}}^2)\exp(iQ_z d_{\text{avg.,B}})]} \ , \quad \text{(S7)}$$

where f_{H_2O}, $\sigma_{0,B}$, and $z_{0,B}$ are the atomic scattering factor for water, Debye-Waller like distribution width, and the first layer position of the bulk water, respectively; $\sigma_{\text{avg.,B}}$ and $d_{\text{avg.,B}}$ are the uncertainties in the spacing of layered water and the layer spacing, respectively. Equation (S7) is capable of modeling a range of the bulk water structures near a solid surface from highly-ordered layered water structure to nearly-featureless error-function like profile[2]. The details of modeling strategy, especially, for the interfacial structure factor, $F_{\text{interface}}(Q_z)$, are specific to individual system; therefore, we will describe them in the subsection follows.

Interfacial Structure Factor Modeling Strategies

For the substrate, the planes beneath the top surface layer initially have a chemical composition of 0.852 Zr, 0.148 Y, and 1.926 O atoms to match the stoichiometry of 8% mole Y_2O_3 in ZrO_2. For the surface relaxation, the lattice constants of the top five unit cells (a_1, a_2, a_3, a_4, and a_5, with a_1 being the top surface unit cell) are allowed to vary independently. With these one-dimensional surface relaxation parameters, the positional parameters for both Zr and O atoms for each unit cell can be estimated. We allow one exception that the relative displacement of O with respect to the metal position in the top layer, Δy, is separately estimated, which turns out to be necessary to reproduce the measured data.

For the interfacial hydration structure, we follow the same strategy applied in an early study (olivine (010)-water interface)[3], in which the solid surface with inherent vacancies is stabilized with adsorbed water. There are three types of interactions between adsorbed water and the terminal surface atoms in this case: vacancy-filling by water, direct metal-water interaction, and hydrogen bonding of the next layer water geometrically constrained by the first metal-hydration layer. Although the detailed mechanisms should be different due to the different chemistries, we surmise that the vacancy filling by water and the direct metal-hydration interaction should have the common effect on the next layer adsorption, i.e., strongly constraining the hydrogen bonding thus resulting in an additional layered water structure.

Earlier study has shown that the Y_2O_3 containing zirconia in contact with water or humid conditions tends to have the alloying agent leaching from the surface and Y^{3+} has much higher

solubility than Zr^{4+} in water[4]. Nevertheless, we do not rule out the possibility of Zr depletion from the substrate. Therefore, we model it in such a way that the top plane of YSZ (110) surface is represented by Zr and O atoms as they are the major components. The occupancies of Occ_{Zr} and Occ_{O} for Zr and O atoms are assumed to fulfill the stoichiometry of ZrO_2 for the simplicity. With possible metal depletion from the top surface, the vacancy sites caused by metal and accompanying oxygen depletion are expected to be filled by water species. Since oxygen and water has similar electron density, we model the vacancy filling water with oxygen atoms for the simplicity. From the assumed stoichiometry of ZrO_2, the occupancy of the O atoms therefore is constrained to be the twice of the Zr occupancy, i.e. $Occ_{O} = 2\times Occ_{Zr}$. The occupancy of the vacancy filling water (Occ_{H2O}) is then equal to the vacancy sites that originated from the metal, oxygen, and intrinsic vacancies, i.e. $Occ_{H2O} = (1 - Occ_{Zr}) + (2 - Occ_{O}) = 3\times(1 - Occ_{Zr})$. For this particular top layer, the structure factor $F_{\text{Terminal\_layer}}(Q_z)$ is then expressed as:

$$F_{\text{Terminal\_Layer}}(Q_z) = Occ_{Zr} f_{Zr} \exp(iQ_z z_{Zr}) \exp(-\frac{1}{2}Q_z^2 u_{Zr}^2) + Occ_{O} f_{O} \exp(iQ_z z_{O}) \exp(-\frac{1}{2}Q_z^2 u_{O}^2) + Occ_{H2O} f_{O} \exp(iQ_z z_{H2O}) \exp(-\frac{1}{2}Q_z^2 \sigma_0^2). \quad \text{(S8)}$$

f_{Zr} and f_{O} are the atomic scattering factors for Zr and O atoms, respectively. z_{Zr} and z_{O} are the positions of Zr and O atoms on this layer, respectively. Here, the position of the Zr atom is chosen as the center of coordination (i.e., $z = 0$). All other positions are relative to this coordination, e.g., the atomic positions of the relaxed surface layers are recalculated based on the estimated lattice constant parameters (a_1 through a_5). The position of O atoms is independently estimated as mentioned above by Δy. The distribution width of Zr and O atoms turned out to be not sensitive to fit the data, so they are fixed to be the same as the lattice Debye-Waller factors u_{Zr} and u_{O}. The position of the O equivalent vacancy filling water z_{H2O} was found to be strongly co-vary with that of O atoms so that they are set to be identical. Only its vibrational amplitude σ_0 is independently estimated. With these parameterization, the total required number of parameters for this particular layer (originally nine) is substantially reduced to three (Occ_{Zr}, Δy, and σ_0, Table S1), which avoids trivial estimations.

Above the top surface layer, an adsorbed hydration structure at position z_1 with occupancy of N_1 and another adsorbed layer at position z_2 with occupancy of N_2 are added. The composition in these two layers can be either pure water or mixture of water and metal species (i.e., hydrated metals). The Debye-Waller like positional distribution widths for these two layers are σ_1 and σ_2, respectively.

The adsorbed layers are followed by a transient water layer connecting the interfacial water to the bulk water, which is considered still as a part of bulk water. The bulk water structure in the vicinity of the interfacial region is modeled by a layered water structure, which can form a relatively sharp first layer (i.e., the transient water layer) followed by quickly damping oscillations of the layered water structure that converge to featureless bulk water[5]. The position of the first bulk water layer, z_B, the Debye-Waller like distribution width, σ_B, and the average distribution width of the water layers, $\sigma_{bar,B}$, can be estimated. The occupancy and layer spacing of the bulk water are fixed to be 1.84 water molecules per unit cell area of A_{uc} = 18.75 Å^2 and

$d_{avg.,B}$ = 2.99 Å, respectively, based on the known water density of 1 g/cm^3 at room temperature.

Parameters	Values from Best-fit	
	Sample #1	Sample #2
Chi-square (χ^2)	1.159	1.398
Scale Factor (S)	$2.428\times10^6 \pm 1.67\times10^5$	$1.066\times10^7 \pm 2.04\times10^5$
Robinson surface roughness factor (β)	0.108 ± 0.024	0.113 ± 0.009
Lattice constant of the top UC(a_1, Å)	3.638 ± 0.016	3.649 ± 0.010
Lattice constant 2nd top UC(a_2, Å)	3.650 ± 0.003	3.648 ± 0.002
Lattice constant 3rd top UC(a_3, Å)	3.640 ± 0.002	3.640 ± 0.005
Lattice constant 4th top UC(a_4, Å)	3.643 ± 0.002	3.644 ± 0.002
Lattice constant 5th top UC(a_5, Å)	3.642 ± 0.002	3.642 ± 0.002
Lattice constant of the substrate (a_0, Å)	3.642	3.642
Terminal layer Zr occupancy (Occ_{Zr})	0.728 ± 0.063	0.672 ± 0.033
Terminal layer O position relative displacement (Δy, Å)	-0.007 ± 0.052	-0.122 ± 0.025
O-equivalent vacancy-filling H_2O distribution width (σ_0, Å)*2	0.132 ± 0.047	0.107 ± 0.035
1st adsorbed hydration layer $[M(H_2O)_n]^{x+}$ position(z_1, Å)	1.687 ± 0.048*	1.713 ± 0.014*
1st adsorbed hydration layer occupancy (N_1)	0.488 ± 0.085*	0.276± 0.015*
1st adsorbed hydration layer position distribution width (σ_1, Å)	0.485 ± 0.090*	0.213 ± 0.031*
2nd adsorbed H_2O layer position (z_2, Å)	3.439 ± 0.118	3.435 ± 0.069
2nd adsorbed H_2O layer occupancy (N_2)	1.441 ± 0.347	1.124 ± 0.125
2nd adsorbed H_2O layer position distribution width (σ_2, Å)	0.386 ± 0.206	0.438 ± 0.110
1st bulk H_2O layer position($z_{0,B}$, Å)	4.918 ± 1.019	5.420 ± 0.223
1st bulk H_2O layer position displacement ($\sigma_{0,B}$, Å)	1.286 ± 0.369	1.332 ± 0.101
1st bulk H_2O layer vibrational amplitude ($\sigma_{avg.,B}$, Å)	1.146 ± 0.646	1.499 ± 0.286
* The values based on tentative model assuming $[Y(H_2O)_{5.8}]^{3+}$.		

Table S1. Parameters derived from the best-fit model.

For modeling the 1st adsorbed hydration layer, an anomalous X-ray diffraction study showed that Y^{3+} in aqueous solution is surrounded by 8 water molecules[6]. However, theoretical study suggested that Y^{3+} with 5.8-, 6.5- and 8- fold first shell water hydration have free energy local minima within 1 kcal/mol range[7]. The hydration of Y^{3+} near an oxide-water interface should be more complicated than that in bulk water due to the geometrically anisotropic energy landscape across the interface. The difference of 1 kcal/mol in free energy could be readily compensated by the complicated local interactions such as surface lattice relaxation, formation of Y-O bonding with surface O atom, and electrostatic interactions between Y^{3+} and negatively charged vacancies at the surface, etc[8,9]. Therefore, it is reasonable to consider that Y^{3+} can exist in the adsorbed layer as any of these hydration structures. In our model fitting, all three possible cases of $[Y(H_2O)_n]^{3+}$, where n = 5.8, 6.5, and 8, were tested as the initial condition and it turned out that the occupancy of Y^{3+} in this layer varies slightly according to different hydration shells yet the total electron density profiles are almost identical. The hydration structure of Y^{3+} lies in 3-dimensional space, within this modeled 2-dimensional adsorbed layer, Y^{3+} is likely to be surrounded by fewer than 8 water molecules, but with additional coordination extends to oxygen atom on the terminal surface and the water molecule in the 2nd adsorbed hydration layer. We present the result of 5.8-water hydrated Y^{3+} case in Table S1 as a representative of the adsorbed metal configuration. There is other possibility describing the nature of this layer differently. See the main text.

AFM Measurement

Atomic Force Microscopy images (Fig. S1) were taken for both Sample #1 and Sample #2 to show the surface morphologies. The r.m.s roughness as-shown is 2.4 Å for Sample #1 and 1.0 Å for Sample #2, respectively. The relative variation in the r.m.s roughness (i.e., statistical distribution of height) is rather distinct, while the Robinson β factor (an estimation of effect of partial layer occupancy and its exponential propagation) is identical within the estimation errors (Table S1). For the comparison, the physical unit cell height of YSZ (110) lattice is 1.821 Å. The deflection images show certainly different morphological characteristics between two samples within 1 μm x 1 μm field of view.

However, the both images do not show distinct steps, kinks, and ledges over larger scales (not shown here), which is counterintuitive to an expectation from the surface miscut reaching to ~0.9° for both samples observed during the HRXR measurement. The morphologies shown in Fig. S1 are extended without definite boundaries, and this extended non-uniformity in lateral direction is common for both samples.

Specular HRXR is sensitive to atomic correlations sharply aligned to or projected to the normal direction with respect to crystalline layers. As it typically reveals laterally averaged structure, as shown in Fig. 4 for example, the chemical and structural homogeneity is more important than the morphological uniformity to obtain general features of system independent of sample-to-sample variations. Combined together in this study, the two HRXR measurements from different surfaces that have own surface morphology characteristics as AFM shown confirm the general features for YSZ (110)-water interface.

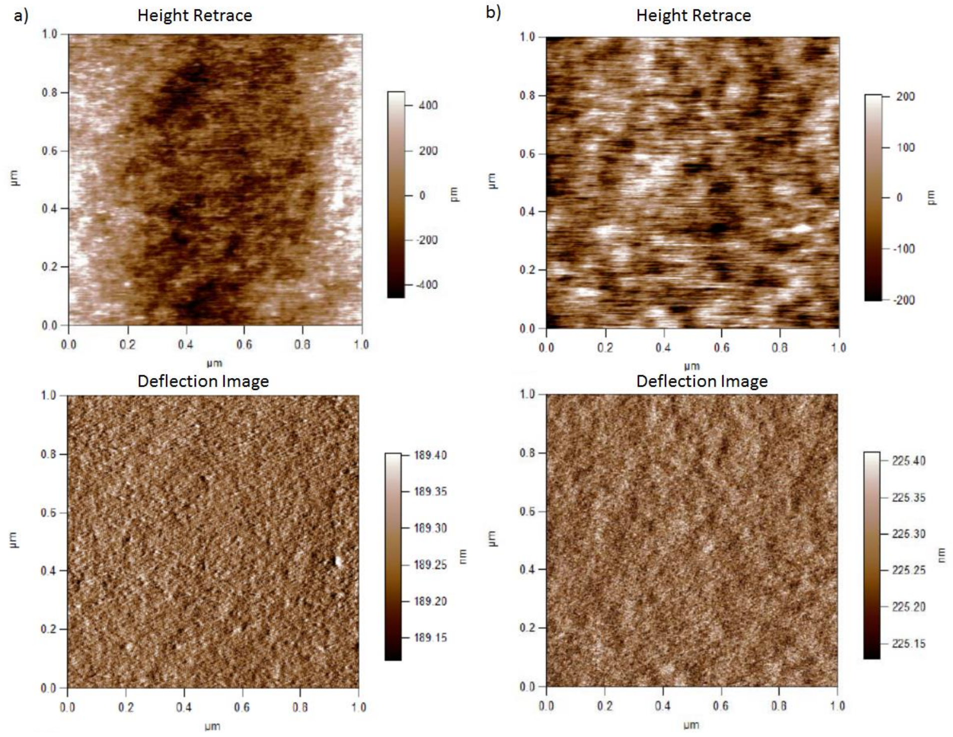

Figure S1. Atomic Force Microscopy images; a) Sample #1 and b) Sample #2.

XPS Measurement for Sample #1

We performed XPS measurement to confirm the surface chemistry. The sample had to be dried and exposed to air before transferred in to vacuum environment so that the analysis does not represent the identical condition with the HRXR measurement. The spectra were measured with increasing Ar sputtering time, which corresponds to a depth profile. The etch level was roughly 0.1 nm/sec with unknown error. The results are shown in Fig. S2.

Among the measured spectra, only O 1s (Fig. S2a) spectra show a recognizable depth dependency in its internal relationship: The spectral profile in overall is almost identical to that found from the reported O 1s spectra for YSZ[10], which includes hydroxide sub-profiles for both ZrO_2 and Y_2O_3. The trend in intensities corresponding to the OH-components (531-534 eV range) is presented in the inset. As the oxide components do not show a distinct depth dependency, the result indicates that the OH-related species exists only near the surface region.

In contrast, the other metal spectra for Zr 3d (Fig. S2c) and Y 3d (Fig. S2d) do not show recognizable depth dependence in their internal relationships. Furthermore, these spectra do not indicate appreciable contributions from own hydroxide components as reported previously[10], although the possibility for minute amount of metal-hydroxide bonding is not completely ruled out. This suggests that the observed decay for OH-related components in O 1s spectra should originate from other source.

The C 1s spectra (Fig. S2b) also show consistent overall profiles with depth, but the majority of peaks consist of adventitious carbons including 284.6 eV and 288.5 eV binding energies, respectively, plus some weaker contributions at around 286 eV. These binding energies are known typical for adventitious carbon[11]. The characteristic methanol binding energy (287.4 eV)[12], which could remain at the surface as a remnant of methanol cleaning, is not recognized significantly.

Based on all information together, the source of decaying hydroxide observed in O 1s spectra is not solely from metal-hydroxide bonding on the surface but more likely interfered by hydroxyl ions in the adventitious hydrocarbons, which also decay in the total amount with increasing sputtering time. And also, there is no recognizable evidence for existence of methanol at the surface, which is known strongly adsorbed on zirconia surface therefore concerned for the possible contamination during the acetone-methanol-water cleaning procedure and hindrance to the interfacial hydration processes. The result confirms consistent YSZ composition over 2 nm range from the top surface and no significant possibility of methanol adsorption during the HRXR measurement.

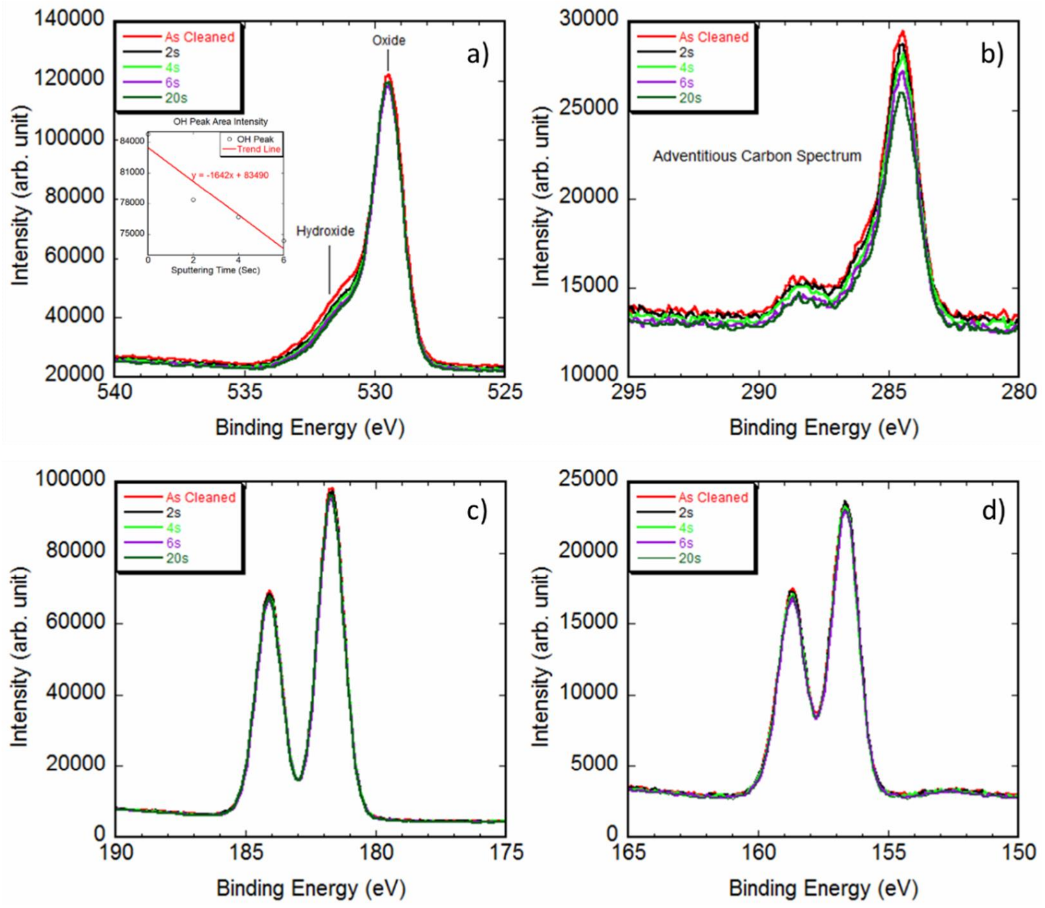

Figure S2. X-ray photoelectron spectroscopy for Sample #1. YSZ (110) single crystal substrate XPS spectra with increasing Ar sputtering time: a) O 1*s* with inset displaying the decrease of hydroxide (OH) peak area after background subtraction and normalization by atomic sensitivity factor, b) C 1*s* showing adventitious carbon, c) Zr 3*d*, and d) Y 3*d*.

References

1 Robinson, I. K. Crystal Truncation Rods and Surface-Roughness. *Phys Rev B* **33**, 3830-3836, doi:DOI 10.1103/PhysRevB.33.3830 (1986).

2 Magnussen, O. M. *et al.* X-Ray Reflectivity Measurements of Surface Layering in Liquid Mercury. *Phys. Rev. Lett.* **74**, 4444-4447, doi:DOI 10.1103/PhysRevLett.74.4444 (1995).

3 Yan, H. P. *et al.* Termination and hydration of forsteritic olivine (010) surface. *Geochim Cosmochim Ac* **145**, 268-280, doi:DOI 10.1016/j.gca.2014.09.005 (2014).

4 Adair, J. H., Krarup, H. G., Venigalla, S. & Tsukada, T. A Review of the Aqueous Chemistry of the Zirconium - Water System to 200°C. *MRS Proceedings* **432**, 101-112, doi:10.1557/PROC-431-101 (1996).

5 Fenter, P. A. X-ray reflectivity as a probe of mineral-fluid interfaces: A user guide. *Rev Mineral Geochem* **49**, 149-220, doi:DOI 10.2138/gsrmg.49.1.149 (2002).

6 Ramos, S., Neilson, G. W., Barnes, A. C. & Mazuelas, A. An anomalous X-ray diffraction study of yttrium(III) hydration. *J Phys Chem B* **105**, 2694-2698 (2001).

7 Ikeda, T., Hirata, M. & Kimura, T. Hydration structure of Y^{3+} and La^{3+} compared: An application of metadynamics. *J Chem Phys* **122**, 244507 (2005).

8 Zhang, Z. *et al.* Ion adsorption at the rutile-water interface: Linking molecular and macroscopic properties. *Langmuir* **20**, 4954-4969 (2004).

9 Lee, S. S., Schmidt, M., Laanait, N., Sturchio, N. C. & Fenter, P. Investigation of Structure, Adsorption Free Energy, and Overcharging Behavior of Trivalent Yttrium Adsorbed at the Muscovite (001)-Water Interface. *J Phys Chem C* **117**, 23738-23749 (2013).

10 Majumdar, D. & Chatterjee, D. X-Ray Photoelectron Spectroscopic Studies on Yttria, Zirconia, and Yttria-Stabilized Zirconia. *J Appl Phys* **70**, 988-992 (1991).

11 *XPS Reference - Carbon*, <http://xpssimplified.com/elements/carbon.php> (2013).

12 Bovet, N., Yang, M., Javadi, M. S. & Stipp, S. L. S. Interaction of alcohols with the calcite surface. *Physical Chemistry Chemical Physics* **17**, 3490-3496, doi:10.1039/C4CP05235H (2015).
